# Large Lipoma of the Mouth Floor

**DOI:** 10.7759/cureus.18420

**Published:** 2021-10-01

**Authors:** Kaitlin Gibson, Mehdi B Swaid, Christopher Metz

**Affiliations:** 1 Otolaryngology, Ascension Providence Hospital, Detroit, USA; 2 Otolaryngology, Michigan State University College of Osteopathic Medicine, East Lansing, USA

**Keywords:** sublingual lipoma, hypoglossal neuromonitoring, oral lipoma, intraoperative neuromonitoring, benign oral mass

## Abstract

Lipomas are one of the most common benign connective tissue masses in the human body. They rarely cause issues and are typically removed for cosmetic reasons. They rarely appear in the oral cavity though they are common. Thus, only a few sublingual lipoma cases have been reported. We present a case of a male in his 60s who came to our clinic complaining of dysphagia and dysarthria caused by an oral swelling from a right sublingual simple lipoma. It was decided to be removed surgically under general anesthesia, with neuromonitoring of the right hypoglossal nerve. The patient tolerated the surgery without complications and fully recovered with complete resolution of his mass effect symptoms. This case demonstrated the importance of having a wide differential diagnosis of oral lesions, especially in a patient with a complicated medical history. Though it is not used often, the case also demonstrated the neuroprotective effect of intraoperative hypoglossal nerve monitoring during sublingual surgeries.

## Introduction

Lipomas are the most common benign tumors affecting the human body. Generally, lipomas are asymptomatic; they are typically removed for cosmetic purposes or when they impair surrounding tissues causing mechanical dysfunction. They are painless, composed of fat, and typically develop slowly in the proximal extremities or the trunk. They are rarely seen in the maxillofacial or oral regions, though they commonly appear all over the human body. Their overall incidence in the oral cavity is thought to be between 1% and 4.4% [1]. In the oral cavity, 45% of lipomas occur in the buccal mucosa; other common locations include the lip, salivary glands, palate, tongue, and floor of the mouth/sublingual area [2]. Of these occurrences, the incidence of oral lipomas appearing in the floor of the mouth is 10.2% [2]. Thus, the overall incidence of a lipoma growing in the floor of the mouth is 0.1%-0.44% of all lipomas appearing in the body. Lipomas can be classified based on their histological cell composition, presence or absence of encapsulation, and presence or absence of nearby tissue invasion. Simple lipomas make up 53.5% of fatty oral benign lesions [3].

Even though lipomas are benign, slow growing, and generally do not invade surrounding tissues, they can cause issues in the maxillofacial region and oral cavity due to the close complex anatomy of those areas. They typically cause a “mass effect” or entrapment of surrounding musculature, glands, nerves, and dental anatomy [4]. This effect on surrounding anatomy can become bothersome, interfering with mastication, speech, and tongue movement [5,6]. The following case demonstrates a 60-year-old male with a rare presentation of a normally benign lesion.

## Case presentation

**Figure 1:**
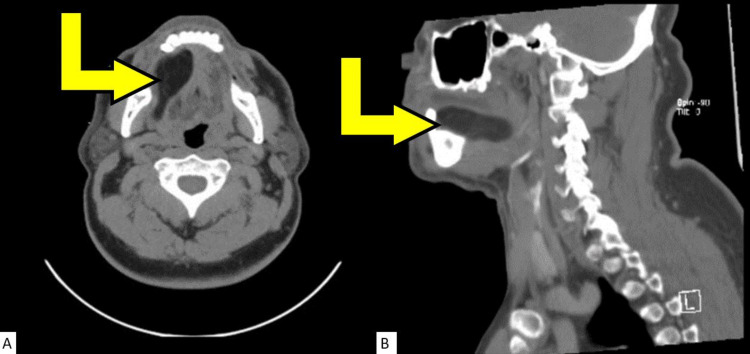
Computed tomography (CT) scans of the patient's head and neck without contrast. The yellow arrows in both images are pointing at the mass in the patient's sublingual area. The low attenuation and smooth borders of the lesion are indicative of a fatty mass. (A) Axial CT scan. (B) Sagittal CT scan.

A 60-year-old African American male came into our outpatient otorhinolaryngology clinic presenting with a painless mass that had been progressively enlarging for the past 10 months on the right side of the floor of the mouth. His main complaints were dysphagia, dysarthria, and oral swelling. Significant medical history included prostate cancer, hyperlipidemia, benign hypertension, type II diabetes mellitus, and tobacco use. Clinical evaluation revealed an intraoral swelling in the right floor of the mouth with smooth benign appearing mucosa. A computed tomographic scan with contrast (Figures 1A, 1B) revealed a smoothly marginated mass within the right sublingual space resulting in a mass effect of the oral cavity musculature, with mild hypertrophy of the palatine tonsils. The bilateral submandibular and parotid glands were unremarkable with evidence of pathological cervical chain lymphadenopathy bilaterally. Retropharyngeal soft tissues appeared within normal limits. 

**Figure 2:**
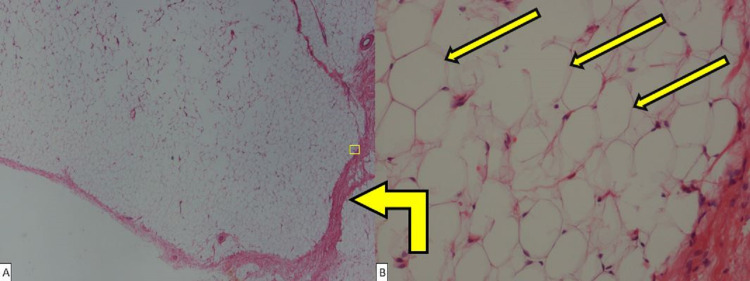
Hematoxylin and eosin stain of fatty appearing sublingual lesion. (A) Low power microscopic image of lesion depicting visible adipocytes, bent yellow arrow pointing to capsule enclosing these cells. The yellow square is a low power view of B. (B) High power microscopic image of adipocytes in the lesion; the three yellow arrows point to individual adipocytes.

Given the clinical presentation, the decision was made to surgically remove the lipoma from the floor of the mouth mass. The location of the mass prompted concern for the risk of damaging the right hypoglossal nerve. To detect and avoid damage to the right hypoglossal nerve, nerve monitoring was utilized during the operation. During the operation, far-field stimulation of the hypoglossal nerve was appreciated. As stimulation of all areas around the base of lipoma did not receive any strong stimulation at any time, the operative field was deemed distant to the right hypoglossal nerve, and dissection of the mass was performed using a combination of blunt and sharp dissection through the sublingual mucosa. The mass was noted to be yellow, homogenous, and encapsulated without invasion into the surrounding musculature and tissues. The excised mass was measured to be 8.5 x 4.0 x 1.0 cm. Hemostasis was achieved, and the incision was closed. Under microscopy, histological features of the mass were constant with features of a simple lipoma without invasion of the surrounding tissues (Figure 2A, 2B). The patient tolerated surgery well, and on follow-up, the patient showed no signs of right hypoglossal nerve damage or residual deficits, and all mass effect symptoms due to the lipoma subsided.

## Discussion

Clinical presentation of floor of mouth masses necessitates thorough physical examination and consideration of a wide range of differentials for diagnosis and treatment. Differential diagnoses for floor of mouth masses consist of broad classifications based on origin including developmental, inflammatory, obstructive, or neoplastic. Developmental masses include dermoid cysts, lipomas, branchial cleft cysts, and thyroglossal duct cysts in which the floor of the mouth is the most common intraoral location for these lesions [7]. Differentiation is important and should be based on a thorough history, physical examination, imaging, and pathological findings. This case illustrates the importance of forming a wide differential when approaching an intraoral mass, especially one belonging to a former smoker and cancer patient. 

Intraoperative neuromonitoring is typically used to protect the facial nerve during parotidectomy and surgeries of the posterior cranial fossa, along with the vagus and recurrent laryngeal nerve during thyroid and parathyroid surgeries. Though there are no guidelines to use neuromonitoring in these cases, it is becoming more commonly used by otolaryngologists to ensure the preservation of these nerves [8]. There is much literature on the protective effects of intraoperative facial and vagus nerve monitoring, but there is very little literature about hypoglossal nerve monitoring being performed [9,10]. However, in this case, neuromonitoring played a key role, helping to prevent intraoperative hypoglossal nerve damage.

## Conclusions

Given the intricate anatomy of the oral region and due to the broad differential for diagnosis, sublingual masses need a comprehensive workup in order to provide the patient with the proper management and treatment. The workup should specifically include a detailed history with family history and social habits, a thorough physical examination of the head and neck, and imaging of the head and neck. Finally, due to the lack of cases and research of intraoperative hypoglossal nerve monitoring, otolaryngologists should consider using intraoperative neuromonitoring of the hypoglossal nerve in sublingual surgeries and then publish their results or conduct a prospective study to provide data on outcomes and morbidity.
